# Comparative Study of Antimicrobial and Antioxidant Potential of *Olea ferruginea* Fruit Extract and Its Mediated Selenium Nanoparticles

**DOI:** 10.3390/molecules27165194

**Published:** 2022-08-15

**Authors:** Hammad Ul Hassan, Naveed Iqbal Raja, Fozia Abasi, Ansar Mehmood, Rahmatullah Qureshi, Zahid Manzoor, Muhammad Shahbaz, Jarosław Proćków

**Affiliations:** 1 Department of Botany, PMAS-Arid-Agriculture University Rawalpindi, Rawalpindi 46000, Pakistan; 2 Department of Botany, University of Poonch Rawalakot, Rawalakot 12350, Pakistan; 3 Department of Pathobiology and Microbiology, PMAS-Arid-Agriculture University Rawalpindi, Rawalpindi 46000, Pakistan; 4 Department of Plant Biology, Institute of Environmental Biology, Wrocław University of Environmental and Life Sciences, Kożuchowska 5b, 51-631 Wrocław, Poland

**Keywords:** SeNPs, biosynthesized, *Olea ferruginea*, antimicrobial andantioxidant activity

## Abstract

Nanotechnology, the science of the recent era, has diverse applications in agriculture. Selenium (Se) is a non-metal and an essential micronutrient for animals and humans. In this study, selenium nanoparticles (SeNPs) were biosynthesized by using *Olea ferruginea* fruit extracts. The size, shape, chemical nature, and identification of functional groups involved in the synthesis of SeNPs were studied by UV-visible spectroscopy, Scanning Electron Microscope (SEM), and Fourier Transform Infra-Red (FTIR) spectrometry. SeNP synthesis was confirmed by an absorption peak at 258 nm by UV-visible spectroscopy. SEM showed that SeNPs were spherical, smooth, and between 60 and 80 nm in size. FTIR spectrometry confirmed the presence of terpenes, alcohols, ketones, aldehydes, and esters as well as phyto-constituents, such as alkaloids and flavonoids, that possibly act as reducing or capping agents of SeNPs in an aqueous solution of *Olea ferruginea*. Antimicrobial activity was examined against bacterial pathogens, such as *Klebsiella pneumonia*, *Escherichia coli*, *Staphylococcus aureus*, and *Staphylococcus epidermitis*, as well as fungal pathogens, such as *Aspergillus niger* and *Fusarium oxysporum*, by using the well-diffusion method. Antioxidant activity was observed using the 2,2-diphenyl-1-picrylhydrazyl (DPPH) assay, ABTs assay, and reducing power assay. At a higher concentration of 400 ppm, biosynthesized SeNPs showed an inhibition zone of 20.5 mm, 20 mm, 21 mm, and 18.5 mm against *Klebsiella pneumonia*, *Escherichia coli*, *Staphylococcus aureus*, and Staphylococcus epidermitis, respectively. Similarly, SeNPs also demonstrated a zone of inhibition against *Aspergillus niger* and *Fusarium oxysporum* of 17.5 and 21 mm, respectively. In contrast to *Olea ferruginea* fruit extracts, *Olea ferruginea*-mediated SeNPs demonstrated strong antimicrobial activity. By performing the DPPH, ABTs, and reducing power assay, SeNPs showed 85.2 ± 0.009, 81.12 ± 0.007, and 80.37 ± 0.0035% radical scavenging potential, respectively. The present study could contribute to the drug development and nutraceutical industries.

## 1. Introduction

Nanotechnology is one of the most interesting and rapidly growing fields of science, agriculture, medicine, and technology [1]. Bio-nanotechnology seeks to develop a reliable and environmentally sustainable method for synthesizing biomaterials at the nanoscale with the help of natural resources [2,3]. Nanoparticles (NPs) are particles that range in size from 1 to 100 nm [1]. They have a variety of biomedical uses because of their higher surface-to-volume ratio and high-surface energies [1,4]. Because of their exceptional qualities, such as their antibacterial and antioxidant activities, biocompatibility, and optical-polarizability, NPs are among the most frequently created and utilized particles [5]. They can be used as catalysts, antimicrobials, antioxidants, memory schemes, and anticancer treatments, among other things [6]. Other applications include drug delivery, tumor hyperthermia, nanocomposites, and medical imaging [7].

Selenium (Se), a non-metal, is an essential macronutrient for both animals and humans where it is needed for the regular physiological and biochemical functioning of human and animal bodies [8]. The daily requirement for Se in the form of dietary supplements for an average adult is 40–300 g. Selenium is a trace element that is present in animals as selenoproteins and is used as a cofactor in numerous enzymatic reactions [9]. However, Se becomes poisonous for people and other living things at higher doses (for example, 3200 g or more per day).Selenium nanoparticles (SeNPs) are one of the many types of NPs that are gaining significant attention due to their high bioavailability, antioxidant activity, antimicrobial qualities, and low toxicity [8,9]. They also have a higher antitumor effect [10]. They are largely being tested for their therapeutic and anticancer activities in the last decade [10,11]. Therefore, there is a need for non-toxic and eco-friendly methods for the synthesis of SeNPs. Recent attempts and research have concentrated on the biological synthesis of SeNPs [12,13], which is both more efficient and less expensive than chemical synthesis.

This study involves the biosynthesis of SeNPs using the fruit extract of *Olea ferruginea* Royle. This plant is a member of the Oleaceae family, and it possesses evergreen leaves. It is commonly called Indian olive or, locally, Kahoo. In Pakistan, the plant is distributed in Pakistan’s Salt range, the Himalayas, the Suleiman ranges, the Hindukush, and the Kalachitta Hills [14]. Although this species can be found anywhere between 500 and 2000 m above sea level, graveyards around 750 m are where it is most commonly found [15]. This species can be used to produce a range of products, such as edible fruits, firewood, olive oil, and fodder. Consequently, the olive tree has economic value [16]. From prehistoric times to the present, *O. ferruginea* has supplied firewood fruit, building wood, fruit, and medicinal items to several cultural groups. *Olea* seeds provide a high content of fat and protein (41%, 37%) [17]. *Olea* species are grown commercially in different countries, including Pakistan, China, India, and Nepal. *Olea* production, along with other crops, has the ability to reduce the high cost of eatable oil imports in Pakistan [18].

Even though many studies have been conducted on the biological synthesis of SeNPs using plant extracts, no studies have been conducted on the synthesis of SeNPs from *O. ferruginea* fruit extract and their comparative antimicrobial and antioxidant activities. Thus, the aims of this work were to (i) biologically synthesize and characterize SeNPs using the fruit extract of *O. ferruginea* and (ii) comparatively evaluate the antimicrobial and antioxidant activities of *O. ferruginea* fruit extract and its mediated SeNPs.

## 2. Results and Discussion

### 2.1. Characterization of SeNPs

#### 2.1.1. UV-Visible Spectroscopy of SeNPs

**Figure 1:**
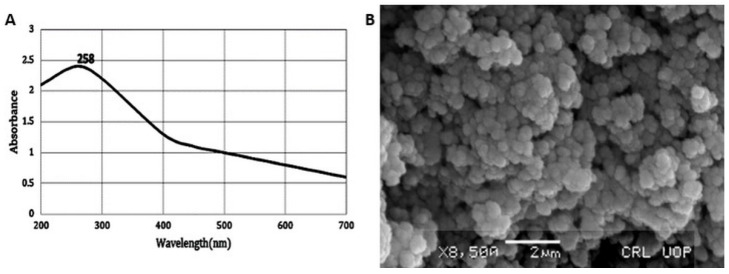
(**A**) UV—Visible Spectrum of SeNPs;(**B**) SEM image of SeNPs.

The formation of SeNPs in the reaction mixture of the plant extract and Na_2_SeO_3_solution was confirmed by UV-visible spectrum in the range of 200–700 nm (Figure 1A). A characteristic absorption peak was recorded at 258 nm due to the change in color from colorless Na_2_SeO_3_to red brick color. Surface plasmon resonance could be the cause of this color change. The characteristic peak at 258 nm confirms the formation of SeNPs. These results are well supported by a previous study in which *Brassica-oleracea*-mediated SeNPs showed a UV-visible-spectra peak at 370 nm [19].

#### 2.1.2. Scanning Electron Microscopy

**Figure 2:**
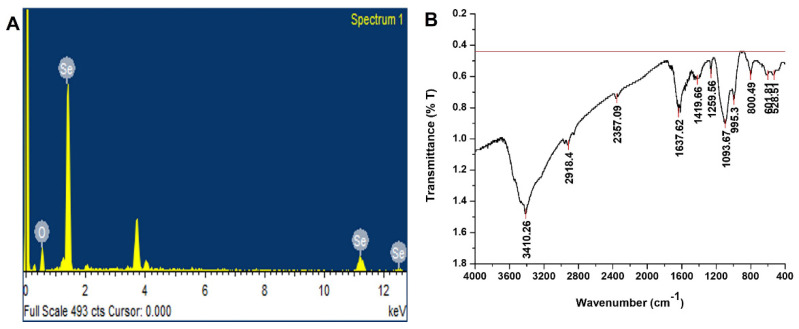
(**A**) EDX Spectrum of SeNPs;(**B**) FTIR spectrum of SeNPs.

The morphology of bio-fabricated SeNPs was studied by SEM. The SEM image of SeNPs revealed that SeNPs were spherical in shape with a smooth surface (Figure 1B). The NPs size was determined to be between 50 and 150 nm. These results correlate with the previous study [19] in which the SEM of *Brassica-oleracea*-mediated SeNPs showed that SeNPs were spherical in shape with a size range between10 and 25 nm. The EDX pattern of SENPs is shown in Figure 2A, which highlights a potent atomic Se signal.

#### 2.1.3. Fourier Transform Infrared Spectroscopy (FT-IR)

An FTIR spectrum of SeNPs was carried out to identify the biomolecules that might be responsible for capping, reducing, and stabilizing SeNPs in aqueous solutions. FTIR spectrum was recorded between the range of 400 and 4000 cm^−1^ (Figure 2B). The FTIR spectrum was recorded and showed stretching and vibrations at wave numbers 528.51, 601.81, 800.49, 995.30, 1093.67, 1259.56, 1419.66, 1637.62, 2357.90, 2918.40, and 3410.26 cm^−1^. These peaks correspond to N-H, -C-H, -C=C, C-O-C, COOH, and C=O and indicate the role of biomolecules in the reduction and formation of SeNPs. The presence of these functional groups on SeNPs predicts phenols, amines, sugars, alcohols, and carboxylic acid as reducing and stabilizing agents of SeNPs. These results are well correlated with the previous study [20].

### 2.2. Antimicrobial Activity of O. ferrugineamediated SeNPs

**Figure 3:**
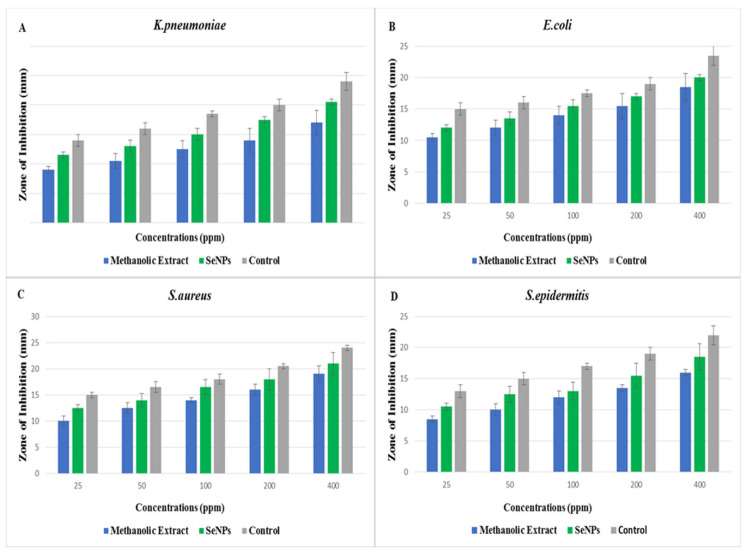
(**A**) Zone of inhibition of *K. pneumoniae*, (**B**) Zone of inhibition of *E. coli. (***C**) Zone of inhibition of *S. aureus*, and (**D**) zone of inhibition of *S. epidermitis*.

The green synthesized SeNPs and *O. ferruginea* plant extract were tested for their antimicrobial activity against different bacterial and fungal strains. The results presented in Figure 3 describe the effect of different concentrations (25, 50, 100, 200, and 400 ppm) of SeNPs and plant extract against phytopathogenic bacteria using the disc-diffusion method. The results showed that every treatment reduced the growth of bacterial strains by creating different sizes of inhibition zones. The higher zones of inhibition, such as 20.5, 20, 21, and 18 mm, were observed against *K. pneumoniae*, *E. coli*, *S. aureus*, and *S. epidermitis*, respectively. However, the plant extract at the same concentration produced inhibition zones of 17, 16.5, 19, and 16 against these bacteria, respectively. SeNPs displayed higher antibacterial activity than plant extracts. Results of the current study are compared to the previous studies [21] in which SeNPs against different bacterial strains have shown different inhibition zones at different concentrations. By comparing current and previous findings, it is found that *O. ferruginea*-mediated SeNPs have great antibacterial potential compared to *O. ferruginea* fruit extract [22].

**Figure 4:**
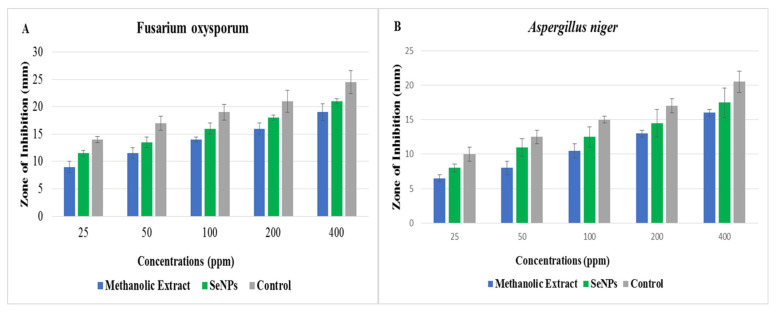
(**A**) Zone of inhibition against *F. oxysporum* and (**B**) Zoneof inhibition against *A. niger*.

The results of different concentrations (25, 50, 100, 200, and 400 ppm) of *O. feruginea-* mediated SeNPs and plant extract against different phytopathogenic fungi are shown in Figure 4. SeNPs showed the higher zone of inhibition against *A. niger* (17.5 mm) and *F. oxysporum* (21 mm) compared to plant extracts, such as 16 and 19 mm against *F. niger* and *F. oxysporum*, respectively. The antibacterial and antifungal activity of SeNPs increased with an increase in the concentration of SeNPs. Results of the present study are paralleled with the previous study [23] in which extract of propolis-mediated SeNPs against fungal strains have shown considerable inhibition zones at different concentrations. Moreover, *O. ferruginea*-mediated SeNPs have more antifungal ability compared to *O. ferruginea* fruit extracts [24].

In past studies, researchers have suggested different mechanisms of antimicrobial activity by NPs. The size of the NPs is one of the major contributors to the antimicrobial activity. The small size of NPs enables them to pass through the cell walls and membranes, resulting in the lysis of cells. Additionally, NPs disrupt the respiratory cycle and ATP generation, halts cell division, and causes microbial cell death [25]. In our study, it was also noted that Gram-positive bacteria were more sensitive to SeNPs than Gram-negative bacteria. This can be explained by the fact that SeNPs have strong electrostatic repulsion toward the lipopolysaccharide and the membrane of Gram-negative bacteria, which is highly negative in nature [26]. However, compared to Gram-negative bacteria, Gram-positive bacteria have a far lower negative charge. Therefore, a larger SeNP deposition on the surface of Gram-positive bacteria may be necessary to cause bacterial mortality. As a result, Gram-negative bacteria frequently display SeNPs resistance.

### 2.3. Antioxidant Activity of SeNPs

**Figure 5:**
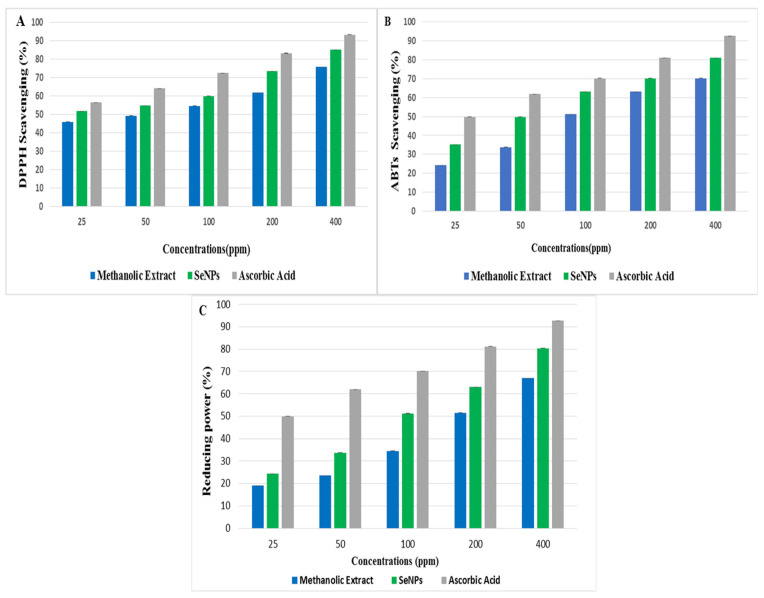
(**A**) DPPH assay of SeNP, (**B**) ABTs assay of SeNPs, and (**C**) reducing power assay of SeNPs.

Antioxidants are agents that inhibit the propagation of an oxidative chain reaction by preventing the oxidation of biologically important macro-molecules. The antioxidant potential of biogenic SeNPs and plant extract was tested using DPPH scavenging, ABTs, and reducing-power assays with ascorbic acid as a control sample and standard. The outcomes of the DPPH experiment revealed that *O. ferruginea*-mediated SeNPs had effectively scavenged the free radicals of DPPH. The free radical scavenging activity increased with the increase in the concentration of SeNPs and plant extract from 25 to 400 ppm. SeNPs, plant extract, and ascorbic acid, respectively, showed maximum DPPH scavenging activities of 85.2, 75.9, and 93.4% at a concentration of 400 ppm (Figure 5A). Present study results are well correlated with the previous study [27] in which the scavenging activity of *Diospyros montana*-mediated SeNPs was 61.12% and ascorbic acid was 99.84%. The antioxidant activity of SeNPs and plant extract against ABTs is shown in Figure 5B. Again, the scavenging activity increased with an increase in plant extract and SeNPs concentrations. SeNPs, plant extract, and ascorbic acid showed maximum ABTs scavenging activity of 81.12, 70.20, and 92.73% at higher concentration of 400 ppm, respectively. Present study results are supported by a previous study [28] in which the ABTS scavenging activity of tree-gum-stabilized SeNPs was 92.2%. These findings support SeNPs’ antioxidant activity [29].

The antioxidant activity of SeNPs and plant extract by reducing power assay is shown in Figure 5C. Different concentrations from 25 to 400 ppm of plant extract and SeNPs were used. SeNPs, plant extract, and ascorbic acid showed maximum-reducing-power scavenging activity of 80.37, 67, and 92.73 at higher concentration of 400 ppm. It was found that green-synthesized SeNPs have much more reducing potential compared to *O. ferruginea* fruit extracts. SeNPs easily cause the reduction of ferric ions to enhance the antioxidant activity [30].

## 3. Materials and Methods

The synthesis of SNPs was performed in the Nanotechnology and Plant Tissue Culture Laboratory, Department of Botany, PMAS Arid Agriculture University Rawalpindi. Biosynthesized SeNPs were then evaluated for their antimicrobial and antioxidant activity.

### 3.1. Preparation of O. ferruginea Fruit Extract

Fruits of *O. ferruginea* were collected from Dil Jabba Hills Village, Shahpur, District Chakwal, latitude 320 55′29.39″ N and longitude 720 51′11.19″ E Pakistan. The fruits were washed with tap water followed by distilled water to ensure the complete removal of dust or debris. Then, the fruits were ground into small pieces with the help of a mortar and pestle. The ground fruit (40 g) was weighed by using a weighing balance and then transferred to a 500 mL beaker comprising 400 mL of distilled water. The solution was boiled for 20 min. The extract of *O. ferruginea* was then filtered by using Whatman no. 1 filter paper to obtain the transparent solution and was stored at 4 °C for further use to synthesize SeNPs.

### 3.2. Synthesis of SeNPs

**Figure 6:**
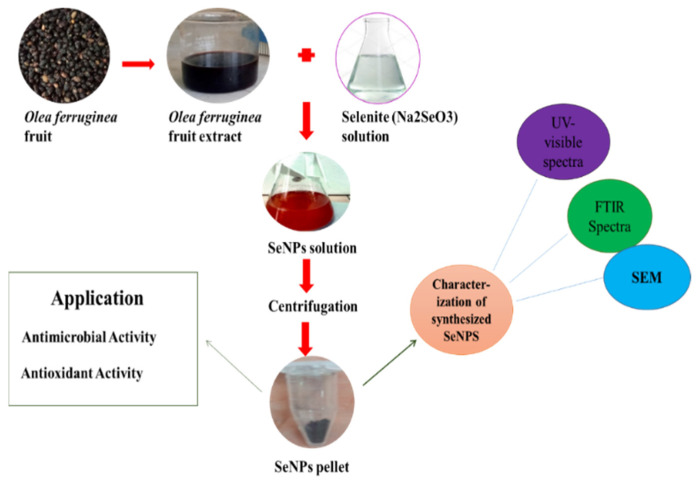
Synthesis of SeNPs from *Olea ferruginea* and their application as antibacterial and antioxidant.

The plant-based green synthesis of SeNPs was achieved by following the protocol reference with slight alteration [31]. A 5 mM solution of Na_2_SeO_3_ was prepared by dissolving 0.348 g of Na_2_SeO_3_salt in 1 L of distilled water. Then, *O. ferruginea* fruit extract was added to it until the color of the reaction mixture changed to brick red, which is characteristic of SeNPs. After the synthesis of SeNPs, the mixture was centrifuged at 14,000 rpm for 15 min for the separation of NPs from the plant extract. SeNPs were then suspended in water. The process of centrifugation was repeated four times to remove all impurities. After purification, we collected the NPs and dried them on filter paper for further experimentation. A protocol for the synthesis of SeNPs is shown in Figure 6.

### 3.3. Preparation of Methnolic Extract

The fruits were powdered using an electric grinder after being air dried at room temperature in the shade. For obtaining methanolic extract, 50 g of powder was soaked with 200 mL of methanol solvent followed by a period of maceration at room temperature for almost 7 days with constant shaking after every 24 h. The mixture was then filtered with using Whatmann filter paper. By using a rotary evaporator, the filtrate was evaporated at low pressure and temperature for obtaining crude extract [32].

### 3.4. Characterization of SeNPs

#### UV-Vis Spectroscopy

For UV-vis spectrometry, the SeNPs were suspended in distilled water followed by 15 min sonication. The suspension of SeNPs was analyzed by recording UV-visible spectrum from 200 to 700 nm by using spectrophotometer (2J1-0004).

### 3.5. SEM of SeNPs

The structural analysis of green synthesized SeNPs was analyzed by using SEM (JSM5910 JEOL, Tokyo, Japan). The scanning electron technique was used at 5 kV, and the magnification of SEM was set up to ×10 k. The sample for analysis was prepared by using a method called the drop-coating method in which carbon-coated copper grid was used. A film of synthesized SeNPs was prepared by dropping the samples on a copper-coated grid. Blotting paper was used to remove the extra solution, and the film of the plant-based SeNPs was dried by placing it under a mercury lamp for 10 min. The surface image of green synthesized SeNPs was analyzed at various magnifications.

### 3.6. FTIR Analysis

An FTIR examination was performed to analyze the different functional groups of compounds from fruit extract of *O. ferruginea* that are involved in the bio-fabrication of SeNPs. SeNPs were centrifuged to obtain them in the form of dried. Examination was carried out in the range of 400–4000 cm^−1^ by using a FTIR spectrometer (NICOLET 6700, Thermo, Waltham, MA, USA) with a resolving power of 0.15 cm^−1^.

### 3.7. Energy Dispersive X-ray (EDX) 

The elemental analysis of the green synthesized SeNPs nanoparticles was also performed at the IST, Islamabad by using an energy-dispersive X-ray (EDX) detector (SIGMA model).

### 3.8. Antimicrobial Activity of O. ferruginea-Mediated SeNPs

#### 3.8.1. Microorganisms

Tested bacterial strains (*E. coli*, *S. aureus*, *S. epidermidis*, *K. pneumoniae*) were obtained from PMAS Arid Agriculture University Rawalpindi, while the tested fungus strains (*A. niger*, *F. oxysporum*) were cultured in Laboratory, Department of Botany, PMAS Arid Agriculture University Rawalpindi.

#### 3.8.2. Culture Media

For the culturing of bacterial species, a nutrient agar medium was used. For the culturing of fungal species, a potato dextrose agar medium was used. Both media were sterilized in an autoclave (HVE-50 HIRAYAMA) for 15 min at 121 °C.

### 3.9. Antibacterial and Antifungal Activity

The disc-diffusion method [32] was used to test the antimicrobial potential of green synthesized SeNPs against four different strains of bacteria (*K. pneumoniae*, *E. coli*, *S. aureus* and *S. epidermitis*) and two strains of fungal species (*A. niger* and *F. oxysporum*). A loop of microorganism was dipped in a sterilized test tube with distilled water. A 1 mL dilution from a test tube for each strain approximately 2 × 10^8^ cfu mL^−1^ was transported in a sterilized petri dish. The nutrient-ager medium and dilution were mixed well in sterilized petri dishes by shaking. For solidification, the petri plates were kept at room temperature. Filter paper discs that have a diameter of 6 mm were sterilized and dipped in a concentration of antibiotics (Streptomycin, Terbinafine) as the control. Methanolic extracts of *O. ferruginea* fruitand different concentrations (25, 50, 100, 200, and 400 ppm) of green-synthesized SeNPs were then placed on petri dishes containing nutrient-ager medium. Petri dishes were then incubated at 37 °C for 24 h for bacterial culture and at 25 °C for 72 h for fungal culture. The entire experiment was carried out in well-sterilized aseptic conditions. A zone of inhibition was measured in mm with the help of a measuring scale.

### 3.10. Antioxidant Activity of O. Ferruginea-Mediated SeNPs

DPPH assay; DPPH radical scavenging method [33] was used to evaluate the antioxidant activity of green-synthesized SeNPs and *O. ferruginea* methanolic fruit extract. A stock solution of DPPH was prepared by dissolving DPPH (7 mg) in 100 mL methanol. Then, 1 mL of SeNPs and plant extracts of various concentrations (25–400 ppm) was mixed with DPPH solution individually. The mixture was then incubated for 30 min in the dark to note the absorbance of blank and sample readings by using UV-visible spectrometer (BioAquarius CE 7250, Cambridge, UK) at the wavelength of λ_max_ 517 nm. Ascorbic acid was used as standard. Radical scavenging activity was measured by the use of the following formula.

% Inhibition = Absorbance of blank − Absorbance of tested sample/Absorbance of control × 100

ABTS scavenging activity. The ABTS radical scavenging activity of SeNPs and plant extract was evaluated [28] with slight modifications. In this method, 7.4 mM ABTS solution was prepared in 2.5 mM potassium persulphate. Then, the solution was incubated in the dark for 12–18 h at room temperature to obtain a stable oxidative. The stock solution of ABTS was incubated because ABTS and potassium per-sulfate reacted with each other stoichiometrically at a ratio of 0.5:1.0. This ultimately resulted in the incomplete oxidation of ABTS. However, the solution of ABTS oxidized immediately. To attain the maximal absorbance, solution must be elapsed for at least 5–6 h in the dark. Furthermore, the radical becomes stable when it is stored at room temperature in the dark for more than 2 days. To obtain an absorbance of 0.4 at 734 nm, the solution was diluted with sodium-phosphate buffer (10 mM and pH is 7.4). Then, 1 mL of SeNPs and plant extracts of various concentrations (25–400 ppm) were mixed with the solution of ABTS. This mixed solution was incubated for 60 min in the dark. For the standard, ascorbic acid was used. Standard methanol was used as a blank. All the absorbances were noted for each sample and standard [34]. At the wavelength 734 nm, absorbance was noted to calculate ABTS scavenging (%) by using the following formula.

ABTS scavenging (%) = (Blank absorbance − sample absorbance/Blank absorbance) × 100

#### 3.10.1. Reducing Power Assay

The reducing power of SeNPs and plant extract was determined by following protocol [35] with slight alterations. The reducing power assay was based on the deduction of Fe^+3^ ions into Fe^+2^ ions in the presence of various antioxidants. Different concentrations (25, 50, 100, 200, and 400 ppm) of SeNPs and plant extract solution were mixed with 2.5 mL of phosphate buffer and 2.5 mL of (1%) potassium ferricyanide. Then, this mixture was incubated for 20 min at 50 °C and it cooled immediately. After this, 2.5 mL of 10% TCA was added to the above-mentioned solution and then the solution was centrifuged for 10 min at 3000 rpm. Then, the collected supernatant was mixed with an equal amount of Millipore Milli-Q water. Finally, 1 mL of 0.1% ferric chloride was added to it with upper layer, and the absorbance was measured with the help of spectrophotometer at wavelength of 700 nm. Ascorbic acid was used as a standard. The % age of reducing power was calculated using the following formula.

Reducing power (%) = (Blank absorbance − sample absorbance/Blank absorbance) × 100(1)

#### 3.10.2. Statistical Analysis

SPSS software was used for the calculation of all data obtained in triplicates. Data are presented in the form of mean and standard error of calculated means.

## 4. Conclusions

The current study demonstrates the simple, environmentally friendly, cost-effective, and economical biofabrication of SeNPs from *O. ferruginea* fruit extracts. The aqueous fruit extract of *O. ferruginea* was discovered to be suitable for the production of SeNPs. The synthesized SeNPs showed a high degree of stability, a negative charge, an amorphous nature, a spherical form, and nano-size. Effective bio-potential uses, such as antioxidant and antimicrobial, have been demonstrated using SeNPs. The typical antioxidant ascorbic acid was found to be less effective than the SeNPs, which have demonstrated strong free radical-scavenging actions. Additionally, SeNPs have demonstrated strong antibacterial action against a variety of foodborne pathogens. Fungi were found to be the most effective target, followed by Gram-positive and Gram-negative bacteria. As a result of these bio-potential effects, SeNPs are uniquely suited for use as antibacterial and antioxidant agents in pharmaceutical, biomedical, and food industries. SeNPs synthesized from *O. ferruginea* fruit extract could be useful to combat many fungal and bacterial diseases as well. Therefore, the study recommends the SeNPs from *O. ferruginea* fruit extract as a cheap, eco-friendly, and biocompatible alternative that can be used in place of harmful fungicides and antibiotics.

## Data Availability

All obtained data are presented in this article.
